# Impact of L-carnitine in narcolepsy treatment: a systematic review on the effectiveness and safety

**DOI:** 10.5935/1984-0063.20220004

**Published:** 2022

**Authors:** Cristina Salles, Maria Clara Freitas, Miguel Meira e Cruz

**Affiliations:** 1 Escola Bahiana de Medicina e Saúde Pública, International Center on Clinical Sleep Medicine and Research - Salvador - Bahia - Brazil.; 2 Centro Cardiovascular da Universidade de Lisboa, Lisbon School of Medicine, Sleep Unit - Lisbon - Lisbon - Portugal.; 3 Faculdade São Leopoldo Mandic, Neuroimune Pain Interface Lab - Campinas - São Paulo - Brazil.

**Keywords:** Acetylcarnitine, Narcolepsy, Pregnancy

## Abstract

**Introduction:**

Studies have shown that narcolepsy patients may present with low serum acylcarnitine levels, demonstrating a dysfunctional beta fatty acid oxidation pathway in these patients.

**Objective:**

Evaluate the therapeutic efficacy of L-carnitine as a treatment for narcolepsy patients.

**Methods:**

This study runned in form of systematic review. The terms used for the search: (“narcolepsy”[MeSH Terms] OR “narcolepsy”[All Fields]) AND (“carnitine”[MeSH Terms] OR “carnitine”[All Fields] OR “l carnitine”[All Fields]). Were included all surveys published until January 2021, with the diagnosis of narcolepsy, that performed drug treatment with I-carnitine. The clinical endpoints of interest were: excessive daytime sleepiness, dissociative REM sleep manifestations: cataplexy, sleep paralysis, hypnagogic hallucinations, and early REM sleep (REM sleep naps, SOREMP).

**Results:**

L-carnitine was found to be well-tolerated and without side effects in all surveys, at dosages ranging from 500 to 510 mg/day. Newborns did not present complications during delivery.

**Conclusion:**

This study corroborates the efficacy and good tolerability of L-carnitine therapy as a treatment for patients with narcolepsy, including during pregnancy.

## INTRODUCTION

The prevalence of narcolepsy is estimated to be between 1/3,300 and 1/5,000, its etiology is still unknown, however most evidence suggests that an acquired immunemediated sporadic condition often occur in patients with genetic-based vulnerabilities.^1^ Narcolepsy is a chronic hypothalamic neurodegenerative disorder and a quite disabling condition, presenting with several sleep-wake cycle related symptoms, such as excessive sleepiness (ES) and dissociative manifestations of REM sleep, such as cataplexy, sleep paralysis, hypnagogic hallucinations, and early REM sleep (REM sleep naps, SOREMP) ^2^.

According to DSM-V, the individual must present symptoms at least three times a week in the last 3 months to be diagnosed with narcolepsy.^3^ In addition, one of the following must be confirmed: hypocretin deficiency; cataplexy episodes occurring several times per month, latency to REM sleep less than 15 minutes or two or more early REM sleep periods (SOREMPs) and average sleep latency less than 8 minutes.^3^

Two forms of narcolepsy are acknowledged: type 1 narcolepsy (NT1) and type 2 narcolepsy, which were updated from the anterior classification where narcolepsy figured out according to the existence or absence of cataplexy, r^4^. From the 3^rd^ edition of the International Classification of Sleep Disorders (ICSD-3), patient with NT1 will clinically present with excessive daytime sleepiness, cataplexy, hypnagogic hallucinations, and sleep paralysis. NT1 is related to important neurons in the hypothalamus, which are responsible for the production of orexin (hypocretin), a neuropeptide associated with wakefulness. Patients with NT2 do not present with cataplexy, although they present most of the symptoms that characterize NT1 ^4^.

Most treatments for narcolepsy focus on improving the sleep-wake cycle, with special attention to daytime performance^1^. The most important goals are reducing excessive daytime sleepiness (EDS) and cataplexy. Narcolepsy causes marked impairment in the patients’ quality of life, also impacting school and work performance, along with psychosocial difficulties ^5,6^. Non-pharmacological management for control of narcolepsy symptoms should be considered as first alternative of treatment; planned daytime naps are recommended to improve the immediate subjective and objective sleepiness, both in patients with narcolepsy who have not received drugs and in those who are being medicated with stimulants, at any age ^1^. In addition to programmed naps, sleep hygiene, a balanced diet, and physical activity are recommended ^7^. Cognitive behavioral therapy, when indicated for patients with narcolepsy, has as its focus on the resolution of symptoms, identifying and modifying dysfunctional patterns of thought that has a negative influence on behavior and emotions^8^.

Nocturnal sleep is significantly disturbed in at least 65% of patients with narcolepsy. Sleep paralysis and hypnagogic / hypnopompic hallucinations are reported by approximately 50% of patients with narcolepsy^1^. EDS is usually the most notable disabling symptom^1^. Stimulants such as methylphenidate, modafinil, amphetamines and solriamfetol can increase heart rate and blood pressure, with a risk of systemic hypertension^1^. Cataplexy is a pathognomonic symptom of narcolepsy, which is reported by 60% to 70% of all patients with narcolepsy. In moderate to severe cataplexy, pharmacological treatment is usually required (serotonin–norepinephrine reuptake inhibitors such as venlafaxine and sodium oxybate). Antidepressants and hypnosedatives have been used to treat EDS and cataplexy associated with narcolepsy.^9^ Tricyclic antidepressants have been described, since 1960, as being able to improve the symptoms of cataplexy, although no randomized controlled trials have been conducted to confirm efficacy ^10^. Intolerable side effects are common, as is expected a rebound cataplexy, once the drug is withdrawn^9^. No antidepressant is approved by the FDA for treating cataplexy ^10^.

We know that the symptoms of narcolepsy persist during pregnancy, however few studies have evaluated the influence of pregnancy on narcolepsy^11^, and one of the important points to be considered is related to the fact that the drugs used in the treatment of narcolepsy are Class C in pregnancy ^12^, and few data are available in the literature on their effects on the development of both fetuses and neonates. Research has shown that patients with narcolepsy can present low serum levels of acylcarnitine, demonstrating a dysfunctional route of beta-oxidation of fatty acids in these patients ^13^. Therefore, the objective of this survey is to evaluate the therapeutic efficacy of L-carnitine as a treatment for patients with narcolepsy.

## MATERIAL AND METHODS

### Search strategy

This systematic review is registered in Prospero under the protocol CRD42021229801. The search of the surveys was between January 2021 and March 2021 on the electronic databases MEDLINE/PubMed, Scielo, Embase, and Cochrane, through the combination of descriptors, including terms of the Medical Subject Headings (MeSH) and the Health Sciences Descriptors, including publications in English and Portuguese: narcolepsy; and L-carnitine. The terms that were used to the search were related to the population of interest and the parameters wanted for the survey: (“narcolepsy”[MeSH Terms] OR “narcolepsy”[All Fields]) AND (“carnitine”[MeSH Terms] OR “carnitine”[All Fields] OR “l carnitine”[All Fields]). References present in the surveys identified by the search strategy were also manually explored to be added to this survey.

### Inclusion criteria

Were included in this systematic review all surveys published up to January 2021 including patients of both sexes diagnosed with narcolepsy who underwent drug treatment with L-carnitine. The clinical outcomes of interest were excessive daytime sleepiness, dissociative manifestations of REM sleep: cataplexy, sleep paralysis, hypnagogic hallucinations, and early REM sleep (naps with REM sleep, SOREMP).

### Identification and selection of the surveys

The authors read the titles and abstracts of each presorted survey from the search in electronic databases and identified only the ones that fulfilled the inclusion criteria. Then, the complete articles were read to ensure that they fulfilled the systematic review criteria. The divergences were discussed by all authors, to respect the inclusion criteria established previously.

### Data extraction

Two authors performed the data collection through a pre-defined search form. The characteristics of interest included: geographical origin, title, type of survey, length of survey, number of participants and average age of participants. Then, data were collected related to dissociative manifestations of REM sleep, such as cataplexy, sleep paralysis, hypnagogic hallucinations and early REM sleep (REM sleep naps, SOREMP); besides these data from pregnant women, the following were also evaluated: type of delivery, gestational week in which the birth occurred, characteristics of the newborn at birth.

## RESULTS

### Identification and survey selection

**Figure 1.**
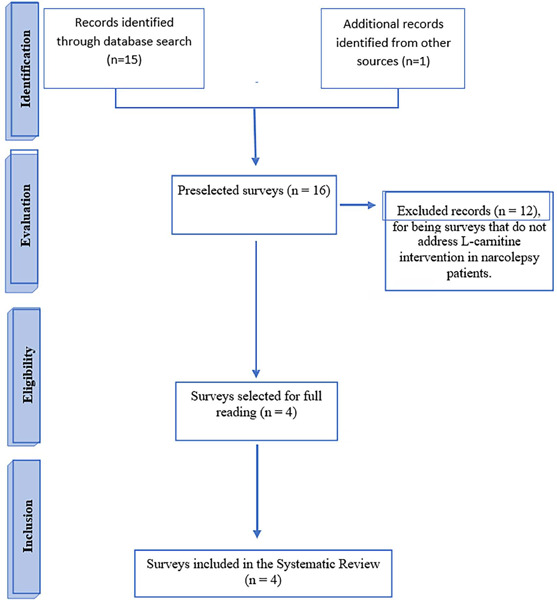
Flowchart of identification, eligibility and inclusion of studies.

Through the search strategy, four (04) registers were identified, including manual search results. After reading the title and abstract, these four (04) articles were selected for full reading. Thus, four (04) articles were selected for systematic review (Figure 1), composing a population of 31 patients with narcolepsy treated with L-carnitine, being 3 of these pregnant women.

Randomized clinical trials and case reports were selected. The general characteristics of the studies included in this systematic review are summarized in Table 1.

**Table 1.** General characteristics of the surveys included in the systematic review

Authors	Year	Origin	Type of survey	N		Survey length
				Male	Female	
Miyagawa et al.,	2013	Tokyo, Japan	Clinical trial	15	13	8 weeks
Williams et al.,	2008	Newark, New Jersey	Case report	0	1	During pregnancy
Romigi et al.,	2015	Rome, Italy	Case report	0	1	During pregnancy
Barros et al.,	2018	São Paulo, Brazil	Case report	0	1	During pregnancy

The samples ranged from 1 to 28 participants (n total = 31), where the youngest age (16 years) was observed in the survey by Williams et al., and the highest mean age, in the survey by Miyagawa et al., where the intervention group had a mean age of 42.17 ± 17 years. This clinical trial was a randomized, double-blind, crossover, placebo-controlled trial lasting 16 weeks. There were two treatment periods lasting 8 weeks each. Five specific visits were established in weeks 0, 4, 8, 12, and 16. Patients were randomly assigned to L-carnitine (510 mg/day), followed by placebo or vice versa. The authors provided the treatment. In Williams et al.’s research, intervention with L-carnitine started before pregnancy and was maintained during the gestational period ^14^. In the research by Romigi et al. and Barros et al., the intervention was performed during the gestational period ^12,15^. These data are shown in Table 2.

**Table 2.** General characteristics of patients with narcolepsy submitted to L-carnitine.

Authors	Year	Average age Years			Narcolepsy type	Comorbity	Other drugs
Miyagawa et al.,	2013	Intervention		Control	With cataplexy	50% obese	NR
		41 + 17		42 + 16			
Williams et al.,	2008		16		With cataplexy	Glutaric aciduria type II	Riboflavin, modafinil, fluoxetine
Romigi et al.,	2015		32		With cataplexy	Obstructive sleep apnea	None
Barros et al.,	2018		34		With cataplexy	Hypothyroidism	Levothyroxine

NR = not referred.

In the research of Miyagawa et al., inclusion criteria were established: age greater than or equal to 15 years and patients who met the diagnostic criteria of the 2nd edition of the International Classification of Sleep Disorders (ICSD-2) for narcolepsy with cataplexy. They adopted the following exclusion criteria: pregnancy, women with suspected pregnancy or breastfeeding; known hypersensitivity to L- carnitine; epilepsy; use of acenocoumarol or other neurological treatments during the research. Thirty patients with narcolepsy were included in this research, however, two (02) patients abandoned the research, because they could not continue the periodic visits pre-established in the research protocol, therefore, 28 patients were included for analysis ^14^. Williams et al. investigated the case of a 16-year-old patient with narcolepsy diagnosed at age 13, treated with modafinil 200 mg/day and fluoxetine 20 mg/day. This patient presented for medical follow-up at the 19th week of pregnancy and according to the data collected, it was observed those four (04) months before pregnancy she was diagnosed with impaired glutaric acid type II. At that time, she presented an altered mental state and urinary tract infection. In this situation, the treatment of choice was L-carnitine and riboflavin, in addition to guidelines for a diet low in fat and protein. During pregnancy, the four (04) medications were maintained ^16^. Romigi et al. reported the case of a 32-year-old woman diagnosed with narcolepsy at age 25 (HLADQB1 * 0602 negative), who presented good therapeutic results using modafinil 400 mg/day, however, this drug was progressively withdrawn, as the patient wished to become pregnant for the first time and, aware of the possibility of teratogenic effects, agreed to treatment with oral L-carnitine at a dosage of 500mg ^15^. Barros et al. reported the case of a 34-year-old patient with hypothyroidism using levothyroxine (75mcg in the morning) and, a diagnosis of narcolepsy type 1, in treatment with modafinil (400 mg/day) associated with citalopram (20 mg/day). This patient wished to become pregnant, so the authors reported the withdrawal of modafinil and citalopram, starting the treatment with L-carnitine (510 mg/day) ^12^.

The data collected on excessive daytime sleepiness and dissociative manifestations of REM sleep, such as cataplexy, sleep paralysis, hypnagogic hallucinations and early REM sleep (REM sleep naps, SOREMP), before being submitted to intervention, are shown in tables 3 and 4, respectively.

**Table 3.** Evaluation of excessive daytime sleepiness in patients with narcolepsy submitted to L-carnitine.

Authors	Year	Epworth Sleepiness Scale		Naps during the day	
Miyagawa et al.,	2013	Intervention	Placebo	Intervention*	Placebo*
		13,7 + 2,9	13,8 + 4	Not referred	Not referred
Williams et al.,	2008	NR		Worsened in pregnancy	
Romigi et al.,	2015	19		Present, with early REM sleep	
Barros et al.,	2018	24		Present, with early REM sleep	

* Number of naps per day; NR: not referred.

**Table 4.** Dissociative REM sleep manifestations in patients with narcolepsy before the use of L-carnitine.

Authors	Year	Cataplexy	Sleep paralysis	Hypnagogic hallucinations
Miyagawa et al.,	2013	Present	Present	Present
Williams et al.,	2008	Present	Not referred	Not referred
Romigi et al.,	2015	Present	Present	Present
		Present		
Barros et al.,	2018	10 episodes per month	Present	Present

The evaluation of excessive daytime sleepiness and dissociative manifestations of REM sleep after treatment with L-carnitine in patients with narcolepsy are shown in tables 5 and 6, respectively.

**Table 5.** Excessive daytime sleepiness after initiation of L-carnitine treatment in patients with narcolepsy.

Authors	Year	Epworth Sleepiness Scale		Naps during the day	
Miyagawa et al.,	2013	Intervention	Placebo	Intervention*	Placebo*
		Decreased	Decreased	Decreased	Decreased
Williams et al.,	2008	Improved		Decreased	
Romigi et al.,	2015	11		Decreased – without early REM sleep	
Barros et al.,	2018	20		Decreased	

**Table 6.** Dissociative REM sleep manifestations in patients with narcolepsy using L-carnitine.

Authors	Year	Cataplexy		Sleep paralysis		Hypnagogic hallucinations	
Miyagawa et al.,	2013	Intervention *	Placebo&	Intervention *	Placebo*	Intervention	Placebo
		0,04 + 0,07	0,02 + 0,04	0,02 + 0,06	0,01 + 0,03	0,02 + 0,06	0,01 + 0,03
Williams et al.,	2008	Number of episodes increased		Not referred		Not referred	
Romigi et al.,	2015	Not referred		Not referred		Not referred	
Barros et al.,	2018	Decreased to 10 episodes per month		Not referred		Not referred	

* Number of episodes per day; NR: not referred.

Miyagawa et al., when comparing the intervention group (L-carnitine) and the placebo group observed a marked reduction in the total siesta time during the day in the first group (L-carnitine: 49±34 min/day sleeping; total siesta time during the day^12^.

Regarding pregnant patients with narcolepsy, it was also evaluated: the type of childbirth, gestational age (week) in which the childbirth occurred, characteristics of the newborn at birth. These are shown in table 7. Williams et al. described that ultrasonography, echocardiography, and cardiotocography of the fetus were within normal limits ^16^. In the survey by Romigi et al., the child was born at 38 weeks, weighing 2,360 g, with Apgar of 7, 8, and 9 ^15^. Barros et al. reported that the patient underwent a cesarean section, with no complications at delivery, where the newborn had Apgar 9/10 and a weight of 3.7Kg ^12^.

**Table 7.** Characteristics of delivery and of the newborn in patients with narcolepsy using L-carnitine.

Authors	Year	Type of birth	Gestational age at the time of delivery	Characteristics of the newborn
Williams et al.,	2008	Elective c-section	37 weeks	No complications
Romigi et al.,	2015	Not referred	39 weeks	No complications
Barros et al.,	2018	C-section	Not referred	No complications

## DISCUSSION

In the present study we could observe that the therapeutic efficacy of L-carnitine as a treatment for patients with narcolepsy in a population of 31 patients showed improvement in the clinical picture of patients regardless of whether they were associated with other drugs. These promising results of L-carnitine may be related to the following factors: about 90% of the individuals who have NTI present a marked reduction or undetectable orexin in the cerebrospinal fluid, i.e., values below 110 pg/ml ^17^. On the other hand, studies in mice with juvenile visceral steatosis that presented primary systemic deficiency of carnitine, would have as a causal factor a defect in renal reabsorption of carnitine ^18^. As for clinical features, these mice showed reduced locomotor activity and fragmented wakefulness, which became more intense in the case of fasting, and were relieved with administration of modafinil ^19^. Tafti et al. ^20^, found that deficient acetyl Co-enzyme A (acyl-CoA) mice had significantly slower theta frequency during REM sleep and lower overexpression of glyoxylase-1. Migaya et al. ^21^, by genotyping narcoleptic individuals, observed that there was an imbalance in Carnitine Palmitoyltransferase 1B (CPT1B). Tafti et al, by performing acetyl-L- carnitine administration, observed a significant improvement in slow theta frequency and glyoxylase-1 overexpression in mutant mice. Based on this report, Migaya et al ^21^, hypothesized that reduced ß-oxidation secondary to lower CPT1B expression, would be able to reduce the hypocretin activity, predisposing to narcolepsy. Kuwajima et al.^22^, conducted a study in mice with juvenile visceral steatosis, they were able to increase the levels of orexin expression by providing the mice with a diet that was rich in L-carnitine.

Through the present study, we were able to observe that none of the newborns presented adverse effects related to the use of L-carnitine during pregnancy. These results are important because all drugs used to treat narcolepsy have a high teratogenic potential, as well as other risks of complications during pregnancy. Among the drugs commonly used for treating narcolepsy^1^, low doses of antidepressants appear to be relatively safe. Damkier et al., evaluated modafinil-related birth defects through national health records of all Danish pregnancies that occurred between 2004 and 2017^23^. Exposure to modafinil was defined as any prescription that took place in the first trimester of pregnancy. Duration of treatment was calculated by dividing the amount prescribed by the standard daily dose, defined as 200 mg. Thus, the authors evaluated 49 pregnant women exposed to modafinil, 963 exposed to methylphenidate, and 828 644 pregnancies not exposed to known teratogens. When analyzing the absolute risks, they found that 12% of women who used modafinil had fetuses with congenital defects. This number was 4.5% for methylphenidate and 3.9% for unexposed. The adjusted ORs were 3.4 (95% CI, 1.2–9.7) comparing modafinil and methylphenidate and, 2.7 (95% CI, 1.1–6.9) comparing modafinil with the group of unexposed pregnant women. Therefore, the authors concluded that in utero exposure to modafinil in the first trimester was significantly associated with an increased risk of congenital malformations compared with methylphenidate or no medication use. Thus, the guidelines recommend discontinuing all medications prior to any planned pregnancy. However, discontinuation will probably result in impaired symptom control^1^. In addition to these pregnancy-related factors, it is important to be aware of the potential for pharmacokinetic interaction^1^of drugs prescribed for narcolepsy control and the concomitant use of oral contraceptives. Most drugs recommended to treat narcolepsy apparently do not present relevant interactions, except for modafinil and pitolisant. The European Medicines Agency recommends dose adjustment and/or additional measures to prevent pregnancy when starting treatment with modafinil or pitolisant in women using low-dose oral contraceptives^1^.

Treatments for narcolepsy aim to improve wakefulness by reducing excessive daytime sleepiness, episodes of cataplexy, hypnagogic hallucinations, and sleep paralysis. Although several CNS stimulant medications are prescribed for narcolepsy patients, to this date, none have achieved 100% efficacy in all patients ^3^. Methylphenidate improves sleep, but has anxiety, headache, and irritability as adverse effects, as does armodafinil; modafinil induces wakefulness, but is not yet safe for prescription in children; sodium oxybate is already FDA approved as a therapy for cataplexy, but should not be combined with other CNS depressants or alcohol. The FDA has also approved pitolisant for narcolepsy.

When women with narcolepsy choose to become pregnant, also comes the need to decide whether or not to maintain medication during pregnancy and lactation ^24^. In choosing to withdraw the medication, the safety of the mother and fetus/baby must be considered, as well as increased absenteeism from work and increased chance of unemployment for working women due to insufficient symptom control^24^. These factors may explain the increased age at first pregnancy for NT1 women and the increased likelihood of single pregnancy^25,26^.

Therefore, the present systematic review allows us to conclude that L-carnitine presented favorable results in narcolepsy control, but not all patients had their condition completely controlled, and there was a need for other interventions in parallel. However, attention is drawn to the need for randomized, placebo and controlled clinical trials, with greater sampling to reach more solid conclusions.
